# Analysis of α-Synuclein Pathology in PINK1 Knockout Rat Brains

**DOI:** 10.3389/fnins.2018.01034

**Published:** 2019-01-09

**Authors:** Rose B. Creed, Matthew S. Goldberg

**Affiliations:** ^1^Center for Neurodegeneration and Experimental Therapeutics, Department of Neurology, The University of Alabama at Birmingham, Birmingham, AL, United States; ^2^Department of Neurobiology, The University of Alabama at Birmingham, Birmingham, AL, United States

**Keywords:** Parkinson’s disease, synuclein, PINK1, Lewy bodies, thioflavin, ubiquitin, inclusions, aggregation

## Abstract

Mutations in PTEN induced kinase 1 (PINK1) cause autosomal recessive Parkinson’s disease (PD). The main pathological hallmarks of PD are loss of dopaminergic neurons in the substantia nigra pars compacta and the formation of protein aggregates containing α-synuclein. Previous studies of PINK1 knockout (PINK1-/-) rats have reported mitochondrial dysfunction, locomotor behavioral deficits, loss of neurons in the substantia nigra and α-synuclein aggregates in various brain regions. We sought to characterize PINK1-/- rats in more detail specifically with respect to α-synuclein pathology because abnormal α-synuclein has been implicated genetically, biophysically and neuropathologically as a mechanism of PD pathogenesis. Moreover, the spontaneous formation of α-synuclein aggregates without α-synuclein overexpression, injection or toxin administration is a rare and important characteristic for an animal model of PD or other synucleinopathies, such as dementia with Lewy bodies and multiple system atrophy. We observed α-synuclein-immunoreactive aggregates in various brain regions of PINK1-/- rats including cortex, thalamus, striatum and ventral midbrain, but nowhere in wild-type (WT) rats. Co-immunofluorescence showed that the α-synuclein-immunoreactive aggregates are both thioflavin S and ubiquitin positive. Many cells in the brains of PINK1-/- rats but not WT rats contained protease-resistant α-synuclein. Total synuclein protein levels were unchanged; however, biochemical fractionation showed a significant shift of α-synuclein from the cytosolic fraction to the synaptic vesicle-enriched fraction of PINK1-/- brain homogenates compared to WT. This data indicates that PINK1 deficiency results in abnormal α-synuclein localization, protease resistance and aggregation *in vivo*. The PINK1-/- rat could be a useful animal model to study the role of abnormal α-synuclein in PD-related neurodegeneration.

## Introduction

Parkinson’s disease (PD) is the most common neurodegenerative movement disorder. Clinically, PD is defined by slowness of movement, rigidity, postural instability, gait abnormalities and tremor. Neuropathologically, PD is predominantly characterized by the loss of dopaminergic neurons in the substantia nigra pars compacta and by the presence of intracellular inclusions, termed Lewy bodies, which are composed mainly of α-synuclein (Spillantini et al., 1998). Point mutations in α-synuclein were the first identified genetic mutations causally linked to PD (Polymeropoulos et al., 1997; Kruger et al., 1998). The subsequent identification of PD-linked α-synuclein gene duplication and triplication mutations indicates that increased expression of WT α-synuclein (which could promote aggregation by increasing α-synuclein protein concentration) is sufficient to cause PD (Singleton et al., 2003). Despite intensive research, the exact role of α-synuclein aggregation in PD remains unclear (von Bohlen Und Halbach, 2004). In an effort to generate a better animal model of PD and a tool for studying potential mechanisms of disease, the Michael J. Fox Foundation for Parkinson’s Research sponsored the generation and initial characterization of PINK1-/- rats as a model of loss-of-function PINK1 mutations causally linked to recessively inherited PD (Valente et al., 2004; Dave et al., 2014). The initial study reported significant motor deficits and age-dependent loss of dopamine neurons in the substantia nigra of PINK1-/- rats (Dave et al., 2014). Subsequent studies of the same line of PINK1-/- rats reported mitochondrial dysfunction, behavioral deficits, loss of neurons in the substantia nigra and locus coeruleus, neurochemical abnormalities and α-synuclein aggregates in various brain regions (Grant et al., 2015; Kelm-Nelson et al., 2015, 2016, 2018; Pultorak et al., 2016; Stauch et al., 2016a,b; Villeneuve et al., 2016a,b). We sought to further characterize PINK1-/- rats specifically with respect to α-synuclein pathology because spontaneous formation of α-synuclein aggregates (without α-synuclein overexpression or injection) is a rare and important feature of PD animal models and because α-synuclein aggregation has been implicated both genetically and biochemically as a mechanism of PD pathogenesis as well as a potential therapeutic target (Goldberg and Lansbury, 2000; Creed and Goldberg, 2018).

## Materials and Methods

### Animals

PINK1-/- rats were obtained from Horizon Discovery and bred to obtain homozygous PINK1-/- and WT Long-Evans controls. Animals were maintained on a 12-h light/dark cycle and were allowed food and water *ad libitum*. This study was carried out in accordance with the recommendations of the NIH Guidelines for the Care and Use of Laboratory Animals. All animal experiments were reviewed and approved in advance by the University of Alabama at Birmingham Institutional Animal Care and Use Committee.

### Immunohistochemistry

Animals were euthanized with CO_2_ and immediately perfused with phosphate-buffered saline (PBS, 0.01 M, pH 7.4). Brains were removed and fixed in 10% formalin overnight at 4°C, then transferred to PBS+30% sucrose and maintained at 4°C 2–3 days for cryoprotection. Brains were frozen and sectioned in the coronal plane at 30 μm thickness using a sled microtome. Sections were collected in multi-well plates with each well containing a series of systematically spaced sections (every 10th section). Entire wells of free-floating sections spanning the disease-relevant regions (cortex, striatum, midbrain and thalamus) were blocked in 1% normal goat serum (NGS) in PBS for 1 h, then incubated in primary antibody (BD Biosciences anti-synuclein #610787 diluted 1:1,000) overnight at 4°C. Sections were washed in PBS and incubated with biotinylated goat anti-mouse secondary antibody for 2 h at room temperature, followed by avidin-biotin peroxidase complex solution (Vector Laboratories ABC Elite) for 2 h at room temperature. Sections were washed in PBS, then developed using DAB chromogen (Vector Laboratories). The location and abundance α-synuclein immunoreactive aggregates were scored by an investigator blinded to genotype and age.

### Proteinase-K Resistant Immunohistochemistry

Immediately prior to DAB immunohistochemistry, free-floating coronal sections were washed with PBS and treated with 2 mg/ml proteinase K (Fisher Scientific 50-751-7334) for 10 min at room temperature. Sections were then washed thoroughly with PBS and analyzed by immunohistochemistry, as described above. Cells containing proteinase K-resistant α-synuclein immunoreactivity were counted by an investigator blinded to genotype using NIS Elements software. Five sections from each animal were analyzed and averaged.

### Thioflavin S Staining

Free-floating coronal sections were mounted on glass slides, allowed to dry overnight, then washed with 70% ethanol followed by 80% ethanol for 1 min each. Slides were then incubated in 1% thioflavin in 80% ethanol for 15 min in the dark, followed by sequential 1-min washes in 80% ethanol and 70% ethanol. For α-synuclein co-immunofluorescence, slides were first treated as above, then incubated with α-synuclein primary antibody (BD Biosciences #610787 diluted 1:250) and analyzed as above.

### Microscopy and Image Analysis

Fluorescence images were collected on a Leica TCS-SP5 laser scanning confocal microscope. Images of DAB stained brain sections were acquired on a Nikon Ni-E microscope and analyzed using NIS Elements software.

### Western Analysis

Brains were harvested and microdissected immediately following euthanasia, then frozen on dry ice and stored at -80°C. Frozen brain tissue samples were thawed on ice and homogenized with a motorized pestle in RIPA buffer (Boston Bio-products, Bp-115) containing protease and phosphatase inhibitors (Sigma P8340). After incubation on ice for 30 min, homogenates were briefly sonicated, then centrifuged at 1,000 ×*g* for 5 min to remove debris. Equal amounts of total protein (measured by Pierce Bradford assay) were mixed with Laemmli buffer, separated by SDS-PAGE and transferred onto 0.2 um PVDF membranes. Membranes were blocked in 1:1 LI-COR Odyssey blocking buffer and TBS with 0.05% Tween 20 (TBS-T) for 1 h at room temperature, then incubated with primary antibody overnight at 4°C, washed, then incubated with LI-COR Odyssey secondary antibodies for 2 h and imaged using a LI-COR Odyssey Scanner.

For biochemical fractionation to obtain cytosolic and synaptic vesicle-enriched fractions according to the methods described by Hallett et al. (2008), cortical tissues from WT and PINK1-/- rats were homogenized in TEVP buffer (10 mM Tris pH 7.5, 5 mM NaF, 1 mM each EDTA, EGTA and Na_3_VO_4_) containing 320 mM sucrose using a dounce homogenizer and centrifuged for 10 min at 800 ×*g* at 4C. The supernatant was centrifuged for 15 min at 9,200 ×*g* at 4°C. Following centrifugation, the supernatant was decanted into a clean eppendorf tube and stored on ice (S2). The pellet was then re-suspended in TEVP buffer containing 35.6 mM sucrose and vortexed gently to dislodge and break the pellet, followed by incubation on ice for 30 min. Vortexed samples were centrifuged for 20 min at 25,000 ×*g* at 4°C, then the supernatants were transferred to clean centrifuge tubes (LS1). The supernatant, along with the S2 supernatants were centrifuged at 165,00 ×*g* for 2 h at 4°C. The resulting supernatant (S3) was used as the cytosolic enriched fraction while the pellet (LP2) was used as the synaptic vesicle enriched fraction.

### Statistical Analysis

Statistical analyses were conducted using Graphpad Prism 7 software. Unpaired Student’s-*t* tests, two-way ANOVA and non-parametric tests, as indicated, were used to determine significance at the 0.05 level.

## Results

### α-Synuclein Immunohistochemistry

**FIGURE 1:**
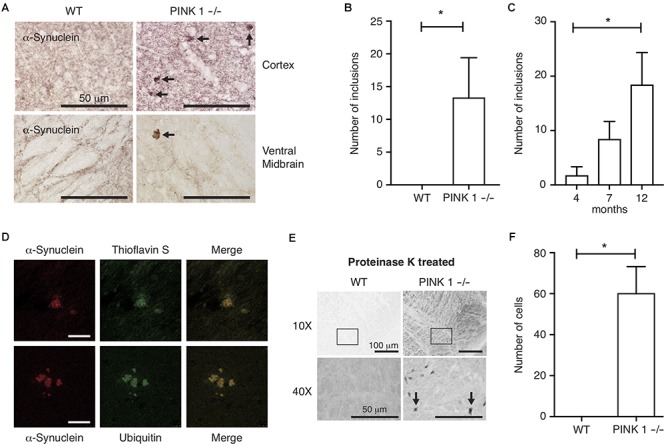
α-synuclein aggregates in PINK1–/– rat brains. **(A)** DAB immunohistochemistry of α-synuclein in the cortex and ventral midbrain of 12-month-old rats. Arrows indicate synuclein-immunoreactive aggregates. **(B)** Mean ± SEM number of α-synuclein inclusions per section, including all brain regions, in (*n* = 17) WT and (*n* = 21) PINK1–/– rats. ^∗^*P* < 0.05 by Mann–Whitney *U*-test. Five systematically spaced sections (every 10th section) were analyzed and averaged for each animal. **(C)** Mean ± SEM number α-synuclein aggregates per section in brains of 4 months (*n* = 5), 7 months (*n* = 6), and 12 months (*n* = 10) PINK1–/– rats. No α-synuclein aggregates were observed in WT rat sections stained and analyzed in parallel (*n* = 5 at age 4 months, *n* = 5 at age 7 months, and *n* = 7 at age 12 months). ^∗^*P* < 0.05 by Tukey’s Multiple Comparison test. **(D)** Co-immunofluorescence of α-synuclein and Thioflavin S or α-synuclein and ubiquitin in the midbrain of PINK1–/– rats at 12 months of age. Scale bar = 10 mm. **(E)** DAB α-synuclein immunohistochemistry in the ventral midbrain of 12-month-old WT and PINK1–/– rats following proteinase K treatment. Top panels are 10× magnification bottom panels are 40× magnification. Arrows in bottom panels indicate cells with proteinase K-resistant α-synuclein. **(F)** Mean ± SEM number of cells per section with proteinase K-resistant α-synuclein immunoreactivity in the midbrain of 3 WT and 3 PINK1–/– rats at age 12 months. Five sections were analyzed and averaged for each animal. ^∗^*P* < 0.05 by Mann–Whitney *U*-test.

Because α-synuclein immunoreactive protein aggregates are pathognomonic for PD, and because genetic, biochemical *in vitro* and *in vivo* studies suggest α-synuclein oligomerization or aggregation is linked to the underlying mechanisms of neurodegeneration in PD, we focused our analysis of PINK1-/- rats on characterizing the pattern and age-dependence of α-synuclein immunoreactivity throughout the brain. DAB immunohistochemistry of coronal sections using α-synuclein-specific antibodies showed a normal pattern of synaptic α-synuclein throughout the neuropil; however, sparse abnormal α-synuclein immunoreactive aggregates were observed throughout the brains of PINK1-/- but not WT rats (Figures 1A,B). Analysis of brain sections from rats at ages 4, 7 and 12 months showed the presence of abnormal α-synuclein immunoreactive aggregates in PINK1-/- rats at all ages, but significantly more aggregates were observed in the older animals (Figure 1C) *P* < 0.05 by Tukey’s Multiple Comparison test. No α-synuclein aggregates were observed in WT rat brain sections at the same ages stained and analyzed in parallel with the PINK1-/- rat brains. To assess the composition of the α-synuclein immunoreactive aggregates in PINK1-/- rats, we conducted immunofluorescence using α-synuclein and ubiquitin-specific antibodies as well as thioflavin S as a well-established fluorescent stain for amyloid. α-Synuclein immunoreactive aggregates showed thioflavin S and ubiquitin staining consistent with amyloid protein aggregates indicative of neurodegenerative disease (Figure 1D).

### Proteinase-K Resistant α-Synuclein in PINK1-/- Rats

α-Synuclein is one of the most abundantly expressed proteins in brain and because α-synuclein is prone to aggregation, we sought to assess the extent to which cells could contain aggregated α-synuclein even if they do not have visible inclusions. To test this, we treated brain sections with proteinase K prior to immunohistochemical analysis, which is a commonly used method to eliminate soluble proteins and to retrieve less accessible epitopes from aggregated proteins, such as aggregated α-synuclein (Beach et al., 2008). Brain sections from 12-month-old WT and PINK1-/- rats were subjected to proteinase K digestion followed by DAB immunohistochemistry with α-synuclein primary antibody. This revealed a large number of cells with proteinase K-resistant α-synuclein in the ventral midbrains of PINK1-/- but not WT rats (Figures 1E,F), as previously reported (Grant et al., 2015). Surprisingly, after proteinase K treatment, we did not observe any α-synuclein aggregates similar to those shown in Figure 1, indicating that those aggregates are labile to proteinase K treatment even though they are thioflavin S-positive.

### α-Synuclein Protein Levels Are Unchanged in PINK1-/- Rats

**FIGURE 2:**
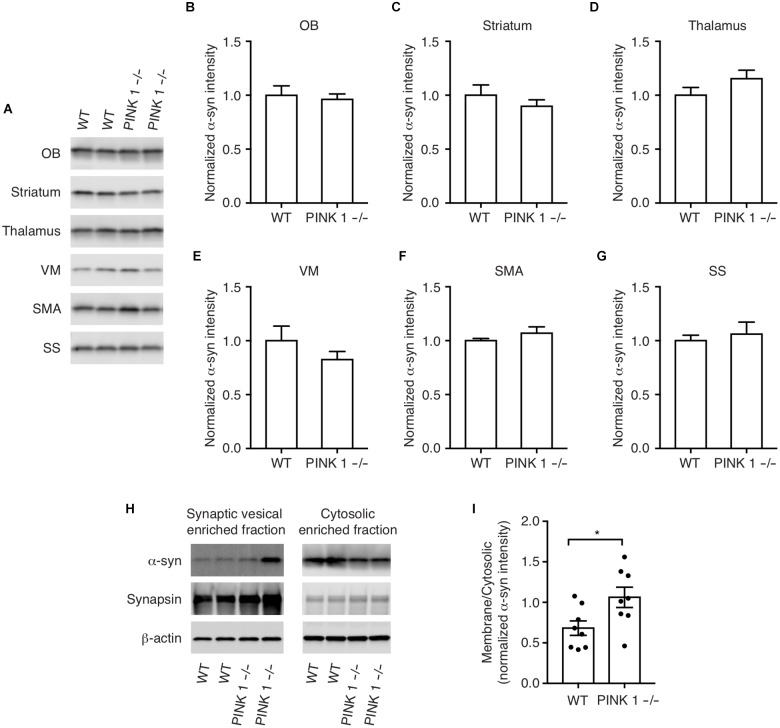
α-synuclein protein levels in WT and PINK1–/– rats. **(A)** Western analysis of α-synuclein in the Olfactory bulb (OB), striatum, thalamus, ventral midbrain (VM), supplementary motor area (SMA), and somatosensory cortex (SS) of WT and PINK1–/– rat brains at age 12 months. **(B–G)** Quantification of western blots from *n* = 8 rats/genotype. Bars represent mean ± SEM. **(H)** Biochemical fractionation and western analysis of cytosolic and synaptic vesicle-enriched fractions of cortex from WT and PINK1–/– rats using antibodies to α-synuclein, synapsin-1, and b-Actin. **(I)** Mean ± SEM ratio of synaptic vesicle bound/cytosolic α-synuclein from *n* = 8 WT and 8 PINK1–/– rats. Asterisk indicates *P* < 0.05 by Student’s *t*-test.

Overexpression of WT α-synuclein can cause the formation of α-synuclein aggregates (Masliah et al., 2000) and α-synuclein gene duplication and triplication mutations cause PD in humans presumably by increased expression of WT α-synuclein (Singleton et al., 2003). This prompted us to examine more carefully the pattern and cellular distribution of endogenous α-synuclein expression in WT and PINK1-/- rats to determine whether the α-synuclein aggregates are possibly caused by increased localized expression of α-synuclein. Western analysis showed no significant differences between WT and PINK1-/- rats in the pattern of α-synuclein immunoreactivity, even within the ventral midbrain (Figures 2A–G).

### PINK1-/- Rats Have Increased Synaptic Vesicle Associated α-Synuclein

α-Synuclein loosely interacts with synaptic vesicle membranes and a shift in α-synuclein membrane interaction, possibly by post-translational modifications, oligomerization, interactions with other proteins, altered lipid composition, or by PD-linked point mutations, can affect the propensity of α-synuclein to aggregate (Dikiy and Eliezer, 2012; Galvagnion et al., 2015, 2016; Ysselstein et al., 2015; Samuel et al., 2016; Nuber et al., 2018). To determine whether PINK1 deficiency alters α-synuclein interaction with synaptic vesicles, we analyzed α-synuclein levels in cytosolic and synaptic vesicle enriched fractions of cortex. We found a relative increase in α-synuclein levels in the synaptic vesicle-enriched fraction of PINK1-/- rat brain compared to WT (Figures 2H,I). This suggests that the observed α-synuclein inclusions may be due to an increase in the interaction of α-synuclein with synaptic vesicles in PINK1-/- rats.

## Discussion

PINK1-/- rats are unusual because α-synuclein immunoreactive aggregates occur spontaneously without synuclein overexpression or administered stresses. In addition to these aggregates, we also detected cells with proteinase K-resistant α-synuclein in the midbrains of the PINK1-/- rats but not WT rats. Our data are consistent with previous reporting of proteinase K-resistant α-synuclein immunoreactivity in PINK1-/- rats (Grant et al., 2015) and α-synuclein aggregates in PINK1-/- rats that could affect neurotransmission underlying motor behaviors (Kelm-Nelson et al., 2016). Although the α-synuclein immunoreactive aggregates we observed in PINK1-/- rats did not resemble Lewy bodies in shape or proteinase K-resistance, we further defined the composition of α-synuclein immunoreactive aggregates by co-localization with thioflavin S and ubiquitin, suggesting that they are composed of amyloid aggregates of α-synuclein, which are characteristics of Lewy bodies (Sakamoto et al., 2002). Even in human brains from PD or Lewy body disease cases, the composition of Lewy bodies and other synuclein aggregates is heterogeneous and this non-uniformity has been proposed to reflect different stages of disease or different phases of synuclein inclusion formation (Sakamoto et al., 2002). Even though total α-synuclein protein levels were unchanged, our biochemical fractionation showed a significant shift of α-synuclein from the cytosolic fraction to the synaptic vesicle-enriched fraction of PINK1-/- brain homogenates compared to WT, which could affect the propensity of α-synuclein to aggregate (Dikiy and Eliezer, 2012; Galvagnion et al., 2015, 2016; Ysselstein et al., 2015; Samuel et al., 2016; Nuber et al., 2018).

## Conclusion

This study establishes the age-dependent accumulation of α-synuclein aggregates that spontaneously form in the brains of PINK1-/- rats. This supports the use of PINK1-/- rats as a unique model to study the role of spontaneous age-dependent α-synuclein aggregation in PD-related neurodegeneration and cellular mechanisms of familial PD.

## Author Contributions

RC designed and conducted the study, analyzed the data and drafted the manuscript. MG analyzed the data and wrote the manuscript. Both authors have read and approved the final manuscript.

## Conflict of Interest Statement

The authors declare that the research was conducted in the absence of any commercial or financial relationships that could be construed as a potential conflict of interest.
